# β2-adrenergic receptor and UCP3 variants modulate the relationship between age and type 2 diabetes mellitus

**DOI:** 10.1186/1471-2350-7-85

**Published:** 2006-12-06

**Authors:** Michele Pinelli, Manuela Giacchetti, Fabio Acquaviva, Sergio Cocozza, Giovanna Donnarumma, Emanuela Lapice, Gabriele Riccardi, Geremia Romano, Olga Vaccaro, Antonella Monticelli

**Affiliations:** 1 Department of Cellular and Molecular Biology and Pathology "A. Califano", University "Federico II", Naples, Italy; 2 Department of Clinical and Experimental Medicine, University "Federico II", Naples, Italy; 3 IEOS, CNR, Naples, Italy

## Abstract

**Background:**

It is widely accepted that Type 2 Diabetes Mellitus (T2DM) and other complex diseases are the product of complex interplay between genetic susceptibility and environmental causes. To cope with such a complexity, all the statistical and conceptual strategies available should be used. The working hypothesis of this study was that two well-known T2DM risk factors could have diverse effect in individuals carrying different genotypes. In particular, our effort was to investigate if a well-defined group of genes, involved in peripheral energy expenditure, could modify the impact of two environmental factors like age and obesity on the risk to develop diabetes. To achieve this aim we exploited a multianalytical approach also using dimensionality reduction strategy and conservative significance correction strategies.

**Methods:**

We collected clinical data and characterised five genetic variants and 2 environmental factors of 342 ambulatory T2DM patients and 305 unrelated non-diabetic controls. To take in account the role of one of the major co-morbidity conditions we stratified the whole sample according to the presence of obesity, over and above the 30 Kg/m^2 ^BMI threshold.

**Results:**

By monofactorial analyses the *ADRB2-27 *Glu27 homozygotes had a lower frequency of diabetes when compared with Gln27 carriers (Odds Ratio (OR) 0.56, 95% Confidence Interval (CI) 0.36 – 0.91). This difference was even more marked in the obese subsample.

Multifactor Dimensionality Reduction method in the non-obese subsample showed an interaction among age, *ADRB2-16 *and *UCP3 *polymorphisms. In individuals that were *UCP3 *T-carriers and *ADRB2-16 *Arg-carriers the OR increased from 1 in the youngest to 10.84 (95% CI 4.54–25.85) in the oldest. On the contrary, in the *ADRB2-16 *GlyGly and *UCP3 *CC double homozygote subjects, the OR for the disease was 1.10 (95% CI 0.53–2.27) in the youngest and 1.61 (95% CI 0.55–4.71) in the oldest.

**Conclusion:**

Although our results should be confirmed by further studies, our data suggests that, when properly evaluated, it is possible to identify genetic factors that could influence the effect of common risk factors.

## Background

Positive selection for allelic variants in genes involved in food utilization, fat deposition and weight gain was useful in past ages when access to food was limiting (thrifty genotype). Such polymorphisms show high frequency and may now lead to obesity and diabetes in the industrialized countries [1]. Among the huge number of genes responsible for the thrifty genotype those influencing the basal metabolic rate, like the β-adrenergic receptors and the uncoupling proteins, seem to play a deciding role [2]. The β-adrenergic receptors (*ADRB*) are involved in thermogenesis regulation and in lipolysis activation [3], whereas the uncoupling proteins (*UCPs*) seem to be the main effectors of heat generation [4].

Several genetic association studies have examined the possible role of *ADRBs *and *UCPs *gene variants with type 2 diabetes mellitus, T2DM, and obesity. However, despite the use of equally valid sample size and statistically significant data, the results obtained in these different studies sometimes conflict. For example, two single nucleotide polymorphisms (SNPs) of *ADRB2 *(Gly16Arg and Gln27Glu) and one of *ADRB1 *(Gly389Arg) were associated with obesity and T2DM in a Swedish and a Finnish study [5-8]. Other studies in Caucasians and an Asian population do not confirm this finding [9-11]. The *UCP2*/*UCP3 *locus is on chromosome 11 and has been linked with diabetes-related phenotypes in human and animal models [12-14]. In particular the *UCP2 *G(-866)A and the *UCP3 *C(-55)T SNPs have been shown to be related to fat distribution and cause an increase in the risk of developing T2DM [15,16]. Again, direct association with T2DM produced contrasting results. In a French sample the *UCP3 *(-55)T variant was associated with a lower risk of developing T2DM. In the same cohort, this variant was associated with the development of atherogenic lipid profile [15].

Type 2 Diabetes mellitus (T2DM) is a complex, multifactorial metabolic disease, characterized by high blood glucose level that results from deficiencies in insulin secretion, insulin action, or both [17]. T2DM is the product of complex interplay between genetic susceptibility and environmental causes like obesity, age, and lifestyle [18].

The results from these studies had variable levels of agreement and were rarely convincing [19-21]. One reason for the lack of replication could involve the presence of several, rare, high penetrance genes or to the presence of several genes that work together, each having a small effect on the disease [22]. Other possible reasons could be the presence of a false-positive association due to population stratification, to the small sample size or the presence of non-linear interactions among genes and environmental factors [22].

To understand complex diseases it is necessary to identify groups of genes that not only interplay with one another but also with environmental factors. The study of only one polymorphism will capture only a few of the total combined effect, therefore it is better to use strategies that can analyse multiple factors together. Multifactor Dimensionality Reduction (MDR) is a method for reducing the dimensionality of multilocus genotype and to improve the identification of polymorphism combinations associated with disease risk. The MDR approach is nonparametric (i.e., no hypothesis about the value of a statistical parameter is made), model-free (i.e., assumes no particular inheritance model), and directly applicable to case-control study designs. MDR is a novel method receiving rising interest in the causes interplay analysis. Both empirical and theoretical studies suggest that MDR has excellent power for identifying high-order gene-gene interactions [23]. The MDR method combines attribute selection, attribute construction, and classification with cross-validation and permutation testing to provide a comprehensive and powerful approach to detecting non-linear interactions [24].

The aetiology of complex disease involves interplay of several causes. The aim of this study is to use multifactor methods to analyse the combined/cumulative effect of genetic and environmental factors on disease such as T2DM. The working hypothesis is that a significant association is more likely to be found when analysing more factors simultaneously. In order to reach this goal, we chose five SNPs already linked to the T2DM and all belonging to a homogeneous group of genes, in which reciprocal interactions are more likely. The SNPs we decided to study are those in genes involved in peripheral energy expenditure and that could be included in the thrifty genotype. In particular we studied two single nucleotide polymorphisms (SNPs) in *ADRB2 *(gly16arg and gln27glu), one in *ADRB1 *(gly389arg), one in *UCP2 *(-866 G/A) and one in *UCP3 *(-55 C/T). We characterised these SNPs in a population from a restricted geographical area, with a homogeneous ethnic background and no recent immigration. We then went on to look for the possible gene-gene and gene-environment interactions comparing the results obtained by the parametric monofactorial analysis with the non-linear interaction-compliant multifactorial analysis (MDR).

## Methods

### Subjects

We studied 342 type 2 diabetic patients, aged 35–70 years, seen at the outpatient diabetic clinic of a health district in the province of Naples over a six-month period. A cohort of 305 unrelated non-diabetic glucose-tolerant control subjects [25] were randomly selected among telephone company employees taking part in a company sponsored health screening. All study participants were Caucasians of Italian origin, unrelated and residents of the same geographical area. The local ethics committee approved the study and informed consent was obtained from all study participants.

Weight, height and waist circumference were measured according to a standard protocol, with participants wearing light clothing and no shoes, BMI was calculated as body weight (in kilograms) divided by squared height (in metres). A blood sample was taken in the fasting state for biochemical measurements. Glucose, triglycerides, total, LDL and HDL cholesterol were measured by standard laboratory methods on fresh plasma. Sitting blood pressure was measured on the right arm after five minutes rest, three readings were taken two minutes apart and the average value was used in the analysis. Use of medication was recorded. Hypertension was defined as blood pressure ≥ 140/90 mmHg or use of antihypertensive medication. For analytical purposes study participants were stratified according to BMI ≥ 30 kg/m^2 ^(obese) or BMI < 30 kg/m^2 ^(non-obese).

### DNA analysis

Genomic DNAs were extracted from 300μl of peripheral blood by using Biorobot EZ1 Qiagen according to manufacturer's protocols. Genotyping was performed using Allele Specific Amplification (ASA) on the Real-Time PCR ABI PRISM 7000 (PE Applied Biosystem, Foster City, CA USA). The following SNPs were tested: Gly389Arg of *ADRB1 *(dbSNP rs1801253), Arg16Gly of *ADRB2 *(dbSNP rs1042713), Gln27Glu of *ADRB2 *(dbSNP rs1042714), G(-866)A of *UCP2 *(dbSNP rs659399) and C(-55)T of *UCP3 *(dbSNP rs1800849). All the oligoprimers were tested by PCR to optimize the melting temperature. Sequence information for all oligonucleotide primers is available as supplementary information [see Additional file 1].

### Statistical analysis

To test for association between genes polymorphisms and the disease, we adopted a multistep strategy. We first performed a parametric monofactorial analysis (mainly performing χ^2 ^tests and computing the Odds Ratios), and then we used the Multifactor Dimensionality Reduction (MDR), a non-parametric multifactorial analysis, to test the combined effect of several genetic and environmental factors. Broadly, MDR outputs the number and the list of factors that could be involved in the disease without giving any additional information on the details of the interaction. To try to explain such interaction we performed a series of monofactorial analyses on the combination of the factors output by MDR analysis. In the latter step, we used a priori and genetic knowledge to reduce the number of analyses. For the monofactorial statistical analyses we considered as significant a p value < 0.05 and to keep a significant result we adopted the Bonferroni multiple testing correction method [26]. Having 3 samples and 5 SNPs we consider 15 monofactorial tests and we obtained a modified p threshold of 0.003. It should be underlined that, in these conditions, this correction method could be considered "highly conservative and may miss real differences" [26]. We estimated the power for our genetic association study using CATS software [27]. To further explore the association between single genetic factors and disease prevalence we calculated the Odds Ratio with 95% Confidence Intervals for the SNP having the greatest χ^2 ^value.

The multifactorial analysis was performed using MDR. MDR is implemented in software (ver. 1.4.1) designed by the Computational Genetics Laboratory (CGL) at Dartmouth Medical School in Lebanon, NH, USA. [24].

For each MDR analysis, the user has to input the population characteristics (disease status, genotypes, and other risk factor data) and the number, *n*, of factors involved in the interaction simultaneously. MDR tries to define the rules involving n factors that best predict the disease status. The output of the analysis is the combination of *n *factors and two values that describe the strength of the association: Prediction Error (PE) and Cross Validation (CV). PE is the percent of subjects whose disease-status is wrongly classified according to the rules, suggesting which portion of the disease risk can be predicted by the output factors. CV represents the self-consistency score of the analysis, computed by performing the same analysis in different subsets of the original data. The latter value measures the likelihood that the result is not incorrect due only to a portion of the dataset [28]. To calculate the p value of the MDR analyses we performed a permutation test. We permutated all the values for each sample and subsample in the disease status column, breaking association between the status, genetic and clinical data. We reiteratively performed this procedure 1000 times and, at the end of each permutation, we performed, again, the MDR analyses for each of the *n *value considered. We collected all PE and CV values for the factors combinations identified. Then we compared the PE and CV values of the real dataset versus the 1000 by *n *values of the permutated dataset. Sorting the permutated dataset for ascending PE and descending CV, we ranked the real result and obtained a value that divided by 1000 by *n *gave us the p value. We performed MDR analyses on three datasets (the whole sample and the two, obese and non-obese, subsample) and therefore we corrected the p value threshold to 0.016 according to the Bonferroni method [26].

The MDR analyses were performed on the whole sample and on the obese and non-obese subsamples. The data entered in the analyses were five SNPs [Gly389Arg of *ADRB1 *(dbSNP rs1801253), Arg16Gly of *ADRB2 *(dbSNP rs1042713), Gln27Glu of *ADRB2 *(dbSNP rs1042714), G(-866)A of *UCP2 *(dbSNP rs659399) and C(-55)T of *UCP3 *(dbSNP rs1800849)] and two non-genetic factors (gender and age) that we considered as environmental factors. It is of relevance that MDR accepts as input discrete variables only. While the first (gender) is by its nature dichotomous, age had to be classified into three main age ranges: 34–53; 54–63 and 64–79. The number, *n*, of interactions tested was 2 and 3.

In addition, to clarify the MDR results we computed the χ^2 ^and the OR (with 95% CI) considering the set of factors selected by MDR. To lessen the number of possible combinations we only analysed dominant models. Again, we corrected the p value by the Bonferroni method.

Finally to confirm the interaction we looked for possible transcript co-regulations among the genes studied. We screened WebQTL [29] a repository of gene expression data of several mouse strains [30].

## Results

In the present study, we analyzed a cohort composed of 305 non-diabetic controls and 342 T2DM patients. Table 1 shows the general characteristics of study participants by diabetes status. According to random selection criterion, as expected diabetic patients were significantly older and more obese, than non-diabetic controls. Also hypertriglyceridemia and hypertension were significantly more frequent in diabetics than non-diabetic participants.

**Table 1:** Clinical and metabolic features of study participants

		**Diabetic Patients**	**Controls**
*No. of subj*.		342	305
*BMI*	*Kg/m*^2^	^a ^31.57 (6.06)	27.25 (4.58)
*Age*	*Years*	^a ^58 (8.34)	54 (6.60)
*Hypertension*	*%*	^a ^75%	56%
*T-CHOL*	*mg/dl*	209 (45.11)	206 (38.13)
*Triglycerides*	*mg/dl*	^a ^159 (108.63)	135 (73.54)
*HDL-CHOL*	*mg/dl*	48 (12.89)	50 (13.68)
*LDL-CHOL*	*mg/dl*	130 (39.48)	130 (35.82)
*Gender*	*% of Males*	^b ^42%	59%

		M (SD)	M (SD)

**a**: t-test *p < 0.001*; **b**: χ^2 ^*p < 0.001*

We genotyped patients and controls for the following SNPs: Gly389Arg of *ADRB1*, Arg16Gly of *ADRB2 *(*ADRB2-16*), Gln27Glu of *ADRB2 *(*ADRB2-27*), G(-866)A of *UCP2 *and C(-55)T of *UCP3*. To consider the co-morbidity between obesity and T2DM the study participants were stratified according to BMI into obese (BMI>30 Kg/m^2^) or non-obese (BMI < 30 Kg/m2). We did not use the BMI as a covariate because we consider obesity as a well-defined disease and not merely as a risk factor for diabetes. Table 2 summarizes genotype frequencies by obesity status.

**Table 2:** Genotype frequencies of the diabetic patients and controls

		**Whole**				**Non-Obese**				**Obese**			
		Controls (305)		Diabetic patients (342)		Controls (228)		Diabetic patients (162)		Controls (77)		Diabetic patients (180)	

		**No.**	***%***	**No.**	***%***	**No.**	***%***	**No.**	***%***	**No.**	***%***	**No.**	***%***

***UCP2***	**A/A**	34	*11*	30	*9*	22	*10*	15	*9*	12	*16*	15	*8*
	**G/A**	124	*41*	145	*42*	95	*42*	70	*43*	29	*38*	75	*42*
	**G/G**	147	*48*	167	*49*	111	*49*	77	*48*	36	*47*	90	*50*
***UCP3***	**C/C**	224	*73*	240	*70*	165	*72*	106	*65*	59	*77*	134	*74*
	**C/T**	78	*26*	94	*27*	61	*27*	49	*30*	17	*22*	45	*25*
	**T/T**	3	*1*	8	*2*	2	*1*	7	*4*	1	*1*	1	*1*
***ADRB1***	**Arg/Arg**	139	*46*	167	*49*	100	*44*	81	*50*	39	*51*	86	*48*
	**Gly/Arg**	134	*44*	136	*40*	103	*45*	63	*39*	31	*40*	73	*41*
	**Gly/Gly**	32	*10*	39	*11*	25	*11*	18	*11*	7	*9*	21	*12*
***ADRB2*-16**	**Arg/Arg**	49	*16*	68	*20*	35	*15*	28	*17*	14	*18*	40	*22*
	**Arg/Gly**	126	*41*	149	*44*	92	*40*	71	*44*	34	*44*	78	*44*
	**Gly/Gly**	130	*43*	124	*36*	101	*44*	63	*39*	29	*38*	61	*34*
***ADRB2*-27**	**Gln/Gln**	135	*44*	172	*50*	99	*43*	72	*44*	36	*47*	100	*56*
	**Gln/Glu**	124	*41*	138	*40*	98	*43*	71	*44*	26	*34*	67	*37*
	**Glu/Glu**	46	*15*	31	*9*	31	*14*	19	*12*	15	*19*	12	*7*

The observed genotype frequencies were in Hardy-Weinberg equilibrium. The frequencies found agree with those previously reported in Caucasians. As monofactorial analyses, we first performed the χ^2 ^test on genotype frequencies using the additive model. The OR was computed between the most protective genotype and the others. No frequency differences were found between patients and controls either in the whole sample or in the obese and non-obese sub-sample for all the polymorphisms. The frequency of the *ADRB2-27 *polymorphism showed the greatest difference between patients and controls, in the whole sample (χ^2 ^= 6.14, p = 0.046) and in the obese group (χ^2 ^9.37, p = 0.009), however the difference was not statistically significant after the correction of the p threshold to 0.003. Complete list of corrected and uncorrected p values are provided as supplementary material [see Additional file 2]. With 80 % power, the minimum detectable relative risk using the sample size studied was 1.51. Further evaluation of this data showed that, in the whole sample, Glu27 homozygotes (Glu27Glu27) of *ADRB2-27 *had a lower frequency of diabetes (Odds Ratio (OR) 0.56, 95% Confidence Interval (CI) 0.36 – 0.91) when compared with Gln27 carriers (Gln27Glu27 or Gln27Gln27). This difference was even more marked in the obese subsample (OR 0.30, 95% CI 0.13–0.67).

According to the frequency data the Population Attributable Risk % (PAR %) for Gln27-carriers could account up to 39% of the diabetes risk in obese subjects and 26% in the whole population.

In the second step, to further study gene-gene and gene-environment interactions we performed the multifactorial analyses. We used Multifactor Dimensionality Reduction (MDR), a nonparametric and genetic model-free approach that uses a data reduction strategy. As quoted in the methods section, five SNPs of interest and the non-genetic factors, age and gender, were tested by the MDR 1.4.1 software for two or three-way interactions.

We performed MDR analyses: on the whole sample, and on each sub-sample (obese and non-obese). For each MDR analysis the range of *n*, the number of interacting factors, was set from 2 to 3. We selected this order of complexity because even in the presence of a statistically significant result more complex interactions could be of no biological meaning.

Table 3 shows, for the sample and for each subsample, the most significant model proposed by MDR having the best value of CV and PE. Interestingly, the best result was obtained by the interaction among age, *ADRB2-16 *and *UCP3 *polymorphisms in the non-obese population (PE = 32.5%).

**Table 3:** MDR Analyses

**Sample**	**Best Model**	**Cross Validation**	**Prediction Error**	***p *value**
**Whole**	**2 **(*Age, Gender*)	10	33.1 %	< 0.001
**Non-obese group**	**3 (*Age, ADRB2-16, UCP3*)**	**10**	**32.5 %**	**< 0.001**
**Obese group**	*-*	-	-	n. s.

**Sample: **sample of the analysis. **Best Model**: number of factors and which factors best predict the disease status in sample. **Cross Validation**: value of the consistency of the model across the 10 repeated test. **Prediction Error**: percent of subjects incorrectly classified following the proposed model. ***p *value**: significant level calculated through a 1000-fold permutation method.

Even if the MDR analysis is able to identify the presence of associations among several factors, its outputs did not give detailed information about the interactions found. MDR output did not specify which age or which polymorphic variant of the two identified genes was high or low risk and furthermore did not describe the three-way interaction.

To carry out this aim, we analysed the combinations of factors (age, *ADRB2-16 *and *UCP3*) gained by MDR results in the non-obese subsample to clarify their single role.

**Figure 1:**
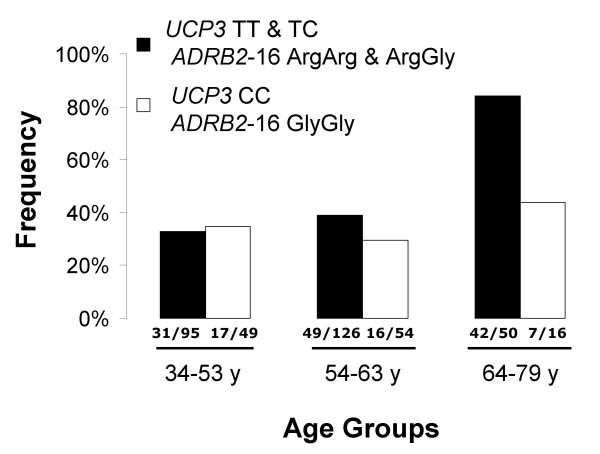
**Percent of T2DM patients for each genotype (*UCP3 *and *ADRB2-16*)and age group in non-obeseparticipants**. On the x-axis the three main age ranges are indicated. The bars indicate the frequencies of non-obese subjects that develop diabetes according to their age range and genotype. Black bars included all the *UCP3 *T and *ARDB2-16 *Arg carriers. White bars represent the double homozygote *UCP3 *CC/*ARDB2-16 *GlyGly. *UCP3 *T/*ARDB2-16 *Arg carriers individuals show an age-related increased frequency of diabetic disease, while double-homozygotes do not. Number of diabetic patients and number of total subjects, divided by a slash, are at the bottom of the bar of each class.

It is well known that the age is a T2DM risk factor and, as expected, performing a χ^2 ^test on a crosstab composed by disease status and age-class, the association was strong (χ^2 ^= 35.24, p = 2.2E-8). Also the OR scaled up from 1 in the youngest group, used as reference group, to 5.76 (95% CI 3.00–11.06) in the oldest group, proving the same conclusion. However, to understand how the two genetic factors could interplay with age, we tested if in each double homozygote there was a different age-related disease risk. We also performed the same analyses on the double complementary genotypes. We found in the double homozygote subjects, *ADRB2-16 *GlyGly and *UCP3 *CC, that the OR was 1.10 (95% CI 0.53–2.27) in the youngest and 1.61 (95% CI 0.55–4.71) in the oldest (χ^2 ^= 1.15, p = n.s.). On the contrary in individuals carrying the other genotypes (*UCP3 *T-carriers and *ADRB2-16 *Arg-carriers) the OR increased from 1 in the youngest to 10.84 (95% CI 4.54–25.85) in the oldest (χ^2 ^= 38.49, p = 4.37E-9). In Figure 1 this difference is expressed by the frequency of the disease in the two different genotypes and in the three age classes. We corrected the p values considering 9 multiple tests resulting in a threshold equal to 0.0056.

## Discussion

We studied gene-gene and gene-environment interactions that could influence Type 2 Diabetes Mellitus. We characterized the genotype of 5 SNPs and collected clinical and anthropometrical information of 647 subjects, including 305 non-diabetic controls and 342 diabetic patients (Table 1). Moreover to consider the frequent co-morbidity of T2DM with obesity we stratified the sample into two distinct subsamples. The framework that suggested this stratification arises from the *common variants/multiple disease *hypothesis [31]. This hypothesis allowed us to suppose that there are genetic variants involved in both T2DM and obesity and genetic variants solely involved in T2DM *without *obesity or in T2DM *with *obesity. We performed monofactorial and multifactorial analyses. In particular we focused our attention on the non-linear interactions using MDR method as statistical tool.

We studied 5 SNPs in 4 different genes involved in basal peripheral metabolism. The gene variants that we choose to study are showed to affect the peripheral energy expenditure. As introduced above, other studies performed on these SNPs failed to provide univocal results. *ADRB2 *(Gly16Arg and Gln27Glu) and *ADRB1 *(Gly389Arg) have both been shown to be associated with obesity and T2DM [5-8]. In particular previous studies demonstrated that *ARDB2 *Glu27 polymorphism influence lifestyles related factors, such as physical exercise [31], diet [32] and weight gain. *UCP2 *G(-866)A and *UCP3 *C(-55)T SNPs have been demonstrated to be related to the fat distribution and the risk of developing T2DM [15,16]. On the other hand, other studies in Europeans [9,10] and in Asians [11] were unable to reproduce these findings. Regarding the studies involving the Asian population, it should be remarked that the frequency of Glu27 allele is very low (Glu27Glu27 less than 1%) [11].

Even if in previous studies these SNPs were variably associated with T2DM, in our study the monofactorial analyses failed to provide any statistical significant association. Indeed, only for the Glu27 *ADRB2-27 *homozygote the OR showed a reduced frequency of the disease in the obese subsample. Also, this finding was less obvious in the whole sample, but not in the non-obese sub-sample. The explanation of this phenomenon is not obvious and it is not clear why this polymorphism could be associated with T2DM in obese but not in non-obese patients. Further, it is relevant to underline that all the SNPs selected are involved in the energy balance, thus it could be possible that this variant is specifically linked to the development of T2DM in obese individuals.

Physiologically *ADRB2 *stimulation induces lipolysis and its polymorphic variants seem to influence this phenomenon. The Gln27Glu *ADRB2-27 *polymorphism seems to alter the agonist-induced receptor down-regulation and thus, indirectly, the overall receptors performance [34]. This polymorphism influences receptor function and therefore the plasma non-esterified fatty acids (NEFA) levels, by a mechanism yet to be fully characterised [5]. The NEFA are key players in the pathogenesis of T2DM, since they could impair insulin production and the insulin sensitivity of the peripheral tissues, stimulate neoglucogenesis and glucose extraction in the liver, and they impair insulin-stimulated glucose metabolism in the muscle [35].

The specific purpose of this work was to investigate the gene-gene and gene-environment interactions, in the hypothesis that small single gene effects could not be detected by monofactorial studies. Furthermore it is our driven hypothesis that genetic factors could modify the response to the environment. We chose SNPs in genes belonging to the same physiological class, to improve the likelihood to detect gene interaction, and age and gender as environmental factors, since age can be considered as the sum on continuous damaging stimuli and the gender represent almost two different endocrinological backgrounds.

In the whole sample MDR detected an interaction between age and gender. This was not an unexpected result, as they were some of the recognized T2DM risk factors and, probably, in this case MDR caught an additive effect. On the contrary, in the obese subsample no result was detected. This output could appear in contrast with some suggestions provided by the monofactorial analyses in which age was significantly associated with the disease in the obese subsample (data not shown) and *ADRB2-27 *Gln carrier resulted to be predominant in diabetic obese individuals. There are several reasons that could explain this result. First, it is possible that there are more than two-factors-interaction in the subsample, reducing the likelihood to detect them, each interaction having a low ability to predict the disease status (high PE) and a low self-consistency in the sample (low CV). Second, it is possible that there are several more complex interactions, even of greater order (n>3), a complexity that we decided not to explore. Third, the association of the age with the disease in the monofactorial analysis was made by a continuous test, the t-test. In the MDR analyses we discretized it in three classes, possibly loosing some association power.

Finally, the MDR in the non-obese subsample suggested a three-factor interaction. This interaction involves age and two SNPs, the *UCP3 *and the *ADRB2-16*. The age is believed to be one of the strongest risk factors for the T2DM [18]. Also *UCP3 *and *ADRB2 *were both previously shown to be involved in T2DM by biological and medical studies [7,15]. In particular, the *ADRB2-16 *variant seems to alter the extent of the agonist-induced lipolysis magnitude [36] and to down regulate the receptor expression [35]. On the other hand the *UCP3 *polymorphism has been associated with T2DM in a French cohort. In this cohort the (-55)T variant seemed to be associated with an atherogenic plasma lipid profile [15]. It is still not clear the pathophysiological role of *UCP3 *in the origin of T2DM. Several mechanisms have been proposed such as influencing the peripheral energy expenditure, protection against oxidative damage, regulation of insulin secretion and fatty-acid or fatty-acid peroxides mitochondrial escape [37].

We did not find, in the literature, evidence to suggest an interaction between the two genes. Through the WebQTL web service we found a strong and inverse correlation between the *ADRB2 *and *UCP3 *transcription levels (Pearsons' r = 0.758 and p = 4.83E-11), in the brain of BDX mouse strain (samples April 2005) [30], proving that the expression of these two genes is co-regulated at least in some tissues. Conceptually, in the thermogenesis function, the Adrenergic Receptors and Uncoupling Proteins work together, having respectively, a regulatory and effector role. The *ADRB2 *activation induces lipolysis and release of NEFA whereas *UCPs *are regulated by NEFA plasma levels and are involved in their metabolism.

Explaining how *UCP3 *and *ADRB2-16 *polymorphisms influence the age-related disease risk is, at the moment, too speculative and beyond the aims of this work. But the understanding that these two polymorphisms may influence the susceptibility to a risk factor strongly suggests the need to further explore the interaction with in vitro and in vivo studies.

## Conclusion

In conclusion, we have presented an association study that used a multianalytical approach. This approach combined monofactorial and multifactorial tests to consider both the main effects and the interactive ones in addition to environmental factors. The results that we obtained seem to confirm that the multifactorial approach and the stratification for co-morbidity are useful tools for the analysis of complex diseases. The consistency of the results that we obtained could be further validated by studying different populations and by using in vitro models.

## Competing interests

The author(s) declare that they have no competing interests.

## Authors' contributions

MG and FA carried out the genotyping; MP performed the statistical analyses and participated to the selection of the genetic variants; SC and AM participated in the design and coordination of the study; EL, GR and GD participated in the clinical characterization of the sample; OV collaborated to the statistical analyses; GRic participated in the coordination of the study. All the authors read and approved the final manuscript.

## Pre-publication history

The pre-publication history for this paper can be accessed here:



## Supplementary Material

Additional File 1 PDF document reporting the oligoprimers sequences. Click here for file

Additional File 2 PDF document reporting the corrected and uncorrected *p *values of each monofactorial analysis. Click here for file
