# Supplementary material for: β2-adrenergic receptor and UCP3 variants modulate the relationship between age and type 2 diabetes mellitus
Source: BMC Med Genet. 2006 Dec 6;7:85. doi: 10.1186/1471-2350-7-85 (PMC1712228; doi:10.1186/1471-2350-7-85)
Supplement: Additional File 1 — PDF document reporting the oligoprimers sequences. [file 1471-2350-7-85-S1.PDF]

# Table S1

| *Gene/SNP* | | *Oligoprimer* |
|---|---|---|
| UCP2 | Reverse: | 5’ GGACTCCGTTTCCTCATTGT 3’ |
| G(-866)A | Forward C: | 5’ GTCCTGTGGGGGTAACTGAC 3’ |
| rs659399 | Forward T: | 5’ GTCCTGTGGGGGTAACTGAT 3’ |
| UCP3 | Reverse: | 5’ GAGAAAGGGGTCTTACCTGTG 3’ |
| C(-55)T | Forward C: | 5’ ATAAGGTTTCAGGTCAGCCC 3’ |
| rs1800849 | Forward T: | 5’ GATAAGGTTTCAGGTCAGCCT 3’ |
| ADRB1 | Reverse: | 5’ CGCCTCTTCGTCTTCTTCAACT 3’ |
| Gly389Arg | Forward C: | 5’ GCGCAGCAGAGCAGTCC 3’ |
| rs1801253 | Forward G: | 5’ GCGCAGCAGAGCAGTCG 3’ |
| ADRB2-16 | Reverse: | 5’ ATGACCAGCACATTGCCA 3’ |
| Arg16Gly | Forward A: | 5’ TTCTTGCTGGCACCCAATA 3’ |
| rs1042713 | Forward G: | 5’ CTTGCTGGCACCCAATG 3’ |
| ADRB2-27 | Reverse: | 5’ GACGCTCGAACTTGGCA 3’ |
| Gln27Glu | Forward C: | 5’ CACGACGTCACGCAGC 3’ |
| rs1042714 | Forward G: | 5’ CACGACGTCACGCAGG 3’ |
