# Supplementary material for: β2-adrenergic receptor and UCP3 variants modulate the relationship between age and type 2 diabetes mellitus
Source: BMC Med Genet. 2006 Dec 6;7:85. doi: 10.1186/1471-2350-7-85 (PMC1712228; doi:10.1186/1471-2350-7-85)
Supplement: Additional File 2 — PDF document reporting the corrected and uncorrected p values of each monofactorial analysis. [file 1471-2350-7-85-S2.PDF]

# Table S2

| | **Uncorrected p values** | | | **Corrected p values** | | |
|---|---|---|---|---|---|---|
| | Whole | Non-obese | Obese | Whole | Non-obese | Obese |
| UCP3 | 0.332 | 0.051 | 0.738 | 0.998 | 0.544 | 1.000 |
| ADRB2-16 | 0.207 | 0.562 | 0.725 | 0.969 | 1.000 | 1.000 |
| UCP2 | 0.591 | 0.954 | 0.220 | 1.000 | 1.000 | 0.976 |
| ADRB2-27 | 0.046 | 0.862 | 0.009 | 0.507 | 1.000 | 0.127 |
| ADRB1 | 0.561 | 0.438 | 0.810 | 1.000 | 1.000 | 1.000 |
